# Factors supporting optimisation of psychotropic deprescribing in people with intellectual disabilities within the UK: a modified Delphi study

**DOI:** 10.3389/fpsyt.2025.1652988

**Published:** 2025-08-19

**Authors:** Danielle Adams, Richard P. Hastings, Ian Maidment, Peter E. Langdon

**Affiliations:** ^1^ Centre for Research in Intellectual and Developmental Disabilities (CIDD), University of Warwick, Coventry, United Kingdom; ^2^ Hertfordshire Partnership University NHS Foundation Trust, Hatfield, United Kingdom; ^3^ Intellectual Disabilities Research Institute (IDRIS), University of Birmingham, Birmingham, United Kingdom; ^4^ College of Health and Life Sciences, Aston University, Birmingham, United Kingdom; ^5^ Birmingham Community Healthcare NHS Foundation Trust, Birmingham, United Kingdom; ^6^ Herefordshire and Worcestershire Health and Care NHS Trust, Worcester, United Kingdom

**Keywords:** intellectual disabilities, Delphi, psychotropic medication, deprescribing, medicines optimisation

## Abstract

Overprescribing psychotropic medication for people with intellectual disabilities increases the risk of adverse effects and has prompted deprescribing initiatives internationally. However, factors that support optimal psychotropic deprescribing in this population remain unclear. The aim of this study is to develop consensus within the UK about factors supporting optimal psychotropic deprescribing using an online Delphi study. A modified Delphi study with two rounds was distributed via Qualtrics^XM^ to a panel of UK healthcare professionals working with people with intellectual disabilities. Thirty-four factors derived from research were presented in round 1 and rated on a 5-point Likert-type scale. Ten factors were presented in round 2 comprising of items not achieving ≥85% consensus in round 1. Participants were able to suggest additional factors in round 1. The key finding was a set of 33 factors supporting optimal psychotropic deprescribing that achieved consensus. Following the round 1 questionnaire, 28 statements reached consensus. Key factors related to attitudes, confidence, conflict resolution, person-centred care, shared decision-making, adherence to guidelines, and mutual learning and support. Following round 2, consensus was reached on five further statements, including two derived from free text responses in round 1. Consensus was reached on 33 factors judged important to promote the safe and effective deprescribing of psychotropic medication for people with intellectual disabilities within the UK. Future practice recommendations should promote equitable and sustainable deprescribing practices informed by experiences of carers and individuals with intellectual disabilities.

## Introduction

Psychotropic medicines act on the brain and are used routinely in psychiatry for the management of a range of mental health conditions (1). These medicines include antipsychotics, antidepressants, anxiolytics and mood stabilisers, which are primarily listed under Chapter 4 “Central Nervous System” in the British National Formulary (BNF) (1). However these medicines are sometimes prescribed off-label, more often than necessary, on a long-term basis, and without regular review for people with intellectual disabilities who have not been diagnosed with a mental health condition, as a way to manage behaviours that challenge (2). The terminology in this area remains controversial reflecting concerns around labelling and the potential for stereotyping individuals (3).The terms “challenging behaviour”, “behaviours that challenge” and “behaviours of concern” are commonly used although currently there is no agreed consensus of appropriate terminology. Throughout this study the term “behaviours that challenge” will be used to refer to a range of behaviours including aggressive, disruptive, or destructive behaviours (4) which can be managed in a variety of additional ways using environmental, behavioural, physical health, psychological and communication interventions (4). The issue of overprescribing psychotropic medicines is further complicated by the increased prevalence of mental illness in this population (5–7), the atypical presentation of mental health conditions in people with intellectual disabilities (5), and diagnostic overshadowing where symptoms arising from physical or mental ill-health are misattributed to a person’s intellectual disability resulting in delays in diagnosis and treatment (8, 9). In practice, this complexity means that some individuals may receive psychotropic medication without a clear diagnostic rationale, while others with genuine mental health needs may remain undiagnosed or undertreated.

Excessive prescribing of psychotropic medication also heightens the risk of side effects such as weight gain and metabolic syndrome, complications commonly linked to “atypical” antipsychotics (10) and often difficult to manage (11, 12). This is especially concerning because people with intellectual disabilities often have poorer health than people without intellectual disabilities and are more likely to have multiple health conditions (2). In response to this issue, NHS England launched the STOMP (Stopping over medication of people with a learning disability, autism or both) programme in 2016 (10). Deprescribing, defined as the reduction or discontinuation of medication, is a key programme component (13).

The STOMP initiative brought the issue of overmedication in people with intellectual disabilities into health and social care policy in 2016 (2, 10, 14) followed by guidelines from the Royal College of Psychiatrists (15) and NICE promoting best practices in psychotropic prescribing for “challenging behaviour” (4). Whilst there are local examples of psychotropic deprescribing in this population (16–18), evidence of STOMP’s wider national impact remains limited (19–22). National data from NHS Digital continue to suggest that psychotropic medicines are still overprescribed for people with intellectual disabilities (20). Although antipsychotic prescribing has fallen slightly in people with intellectual disabilities; the percentage of patients with intellectual disabilities who were prescribed antipsychotics has decreased from 14.4% in 2022–23 to 13.9% in 2023-24, compared to 0.9% in the general population (20), the prescribing of anticonvulsants and antidepressants has risen (21, 22) indicating shifts in prescribing patterns rather perhaps than a reduction in overall psychotropic prescribing.

There is also considerable variability in how prescribers approach psychotropic deprescribing, including differences in the deprescribing process, criteria used to determine suitable individuals for deprescribing and the willingness to deprescribe (23, 24). The way these decisions are made is likely to affect the overall effectiveness of deprescribing interventions (24). Focussing on perspectives of healthcare professionals is important as the successful implementation of complex interventions depends not only on structural change and the effectiveness of the intervention, but also on understanding how attitudes, beliefs and values affect decision-making and impact the behaviour of stakeholders (25, 26). This requires an understanding of the factors maintaining current behaviour as well as barriers and facilitators to the desired change (27). Furthermore, Adams et al.’s review highlighted a significant gap in the inclusion of stakeholder experiences in studies on psychotropic deprescribing (28). There is also a lack of research capturing the experiences of the full range of healthcare professionals involved in multi-disciplinary teams (MDTs) whose views and approaches may impact outcomes. Given these gaps in practice and evidence, a consensus study is needed to identify and prioritise the key factors that support safe, effective, and person-centred deprescribing. By engaging a range of healthcare professionals, a consensus approach can help build shared understanding, reduce variability and inform the development of more consistent and inclusive deprescribing practices.

Recent research (24, 28, 29), has identified factors that may optimise psychotropic deprescribing in people with intellectual disabilities. Further, and considering all medication types, Ghosh et al. (30), completed a systematic review and used meta-ethnography to develop a conceptual framework describing the optimisation of medicines in general with people with intellectual disabilities. The medicines optimisation framework is a person-centred approach to the safe and effective use of medicines to achieve best possible outcomes for individuals (31). It aims to ensure that medicines are used in a way that maximises benefit, minimises harm, and aligns with the individual’s needs and preferences (32). For people with intellectual disabilities, this may involve balancing the lowest effective dose of medication with non-pharmacological approaches, such as behavioural interventions, to manage symptoms or behaviours that challenge. Although this framework provides a valuable synthesis of the literature, it does not specifically address psychotropic deprescribing, nor does it sufficiently capture the perspectives of the full range of clinical staff involved in this process. However, it is relevant as deprescribing medicines is one part of the medicines optimisation process (33).

There is a need to understand the key factors supporting successful psychotropic deprescribing by multi-disciplinary healthcare professionals working with people with intellectual disabilities. MDTs play a central role in medication optimisation in the UK and whilst the composition of these teams may vary between different healthcare providers, they typically include learning disability nurses, psychiatrists, psychologists, speech and language therapists (SLTs), and occupational therapists (OTs) (34). These teams, working across both community and inpatient settings, are typically based within secondary care provider organisations, though they maintain close links with primary care. This study therefore focused on MDTs within secondary care.

We aimed to establish consensus amongst a range of healthcare professionals working in MDTs across the UK supporting individuals with intellectual disabilities about the key factors that promote optimal psychotropic deprescribing for individuals with intellectual disabilities using an online modified Delphi exercise. This structured approach facilitated the engagement of a range of healthcare professionals from MDTs, enabling a comprehensive exploration of their perspectives. Previously published Delphi studies have been used in the development of medicines optimisation tools in this population although not specifically for psychotropic deprescribing (35, 36).

## Methods

### Research design

The Delphi method is an iterative, systematic, structured approach for facilitating a panel of experts to reach consensus on a given topic (37, 38). A heterogeneous panel with differing opinions, skills and perspectives can generate more robust Delphi results (39). Therefore, we sought the opinions of a range of healthcare professionals working in MDTs within secondary care across the UK who are professional experts in the field of intellectual disability.

This modified Delphi study maintained participant anonymity in that panel experts were unaware of each other’s individual responses. However, the researchers had access to all responses to facilitate data analysis and synthesis across rounds. The original Delphi method has been adapted and modified for use in a range of disciplines and covering a wide variety of research designs and aims (40). This current study was carried out in line with reporting standards for Delphi studies in social and health sciences (DELPHISTAR) (38) (**Supplementary Table S1**).

Recent research including a systemic review examining the effects and stakeholder views on psychotropic deprescribing, as well as an online survey of pharmacists’ perspectives (24, 28, 29), had identified factors that may optimise psychotropic deprescribing in people with intellectual disabilities. Further, and considering all medication types, Ghosh et al. (30), completed a systematic review and used meta-ethnography to develop a conceptual framework describing the optimisation of medicines in general with people with intellectual disabilities. Our modified Delphi study was designed to build on findings from this earlier research allowing us to align closely with the existing literature. However, to maintain flexibility, participants were given the opportunity to propose additional factors as free text data in the first round. This approach ensured that the findings would be grounded in existing research providing a more direct contribution to existing evidence whilst also allowing for the inclusion of additional perspectives.

Although the first round of a Delphi is typically a qualitative open-ended round to generate potential items followed by subsequent rounds involving questionnaires (38), we adopted a modified Delphi approach and began by directly rating the importance of the statements derived from previous studies (24, 28–30) given that existing relevant evidence was available to inform statement development. While we did not use an open-ended round to generate items, we included a free-text option in round 1 to allow participants to suggest additional priorities or modifications. This approach enabled us to combine evidence informed content with participant led input. It is important to note that the term “modified Delphi” is used variably in the literature and encompasses a range of adaptations from the classic Delphi model (41). There is no single, standardised methodology of the modified Delphi; it is a flexible framework tailored to the needs and context of each study (41, 42).

### Procedure

A list of factors that optimise psychotropic deprescribing was developed through extracted data from three sources:

1. a conceptual framework describing factors supporting medicines optimisation in people with intellectual disabilities developed using meta-ethnography within a systematic review that also includes a nominal group process (30).
2. a systematic review exploring psychotropic deprescribing in people with intellectual disabilities including stakeholder experiences (24, 28).
3. an online survey of pharmacists’ experiences of deprescribing psychotropic medicines for this patient population (29).

The meta-ethnography (30) identified enablers and barriers to medicines optimisation, while the systematic review (28) and pharmacists’ survey (29) identified those specific to psychotropic deprescribing in people with intellectual disabilities. Together, these sources informed the development of the list of factors that support the optimisation of psychotropic deprescribing.

For clarity and ease of reference in the survey, similar factors were presented with overarching category labels although the categories were not used in the analysis.

Our modified Delphi study comprised two rounds, distributed via Qualtrics^XM^. Whilst a three round design is common in Delphi methodology, we determined after careful review of the results from the second round, that a third round was unlikely to alter consensus. By the end of round 2, the level of agreement on the majority of statements had stabilised, with minimal shifts in ratings compared to round 1. Furthermore, most items had either clearly reached or failed to reach the pre-defined consensus threshold (>85% agreement), and qualitative feedback suggested that participants had reached saturation in their reflections. As such, it was determined that additional rounds would add participant burden without significantly contributing to further insights. This is consistent with other modified Delphi studies in healthcare, where two rounds were often used (43, 44) Two rounds were therefore deemed sufficient to answer the research question. The study was given a favourable ethical opinion by the Humanities and Social Sciences Research Ethics Committee (HSSREC) of the University of Warwick, UK (Reference number: HSSREC 136/23-24). Informed consent was obtained electronically from all individual participants included in the study. At the start of the Qualtrics survey, participants were first presented with eligibility screening questions. Those who met the inclusion criteria were then shown an electronic information sheet and a consent form. Participants were required to confirm consent by selecting a checkbox before proceeding to the survey. If consent was not given, the survey automatically terminated, and entry to the study was not possible. As a Delphi is an iterative process and requires the provision of individualised feedback to each participant via email, anonymity of survey responses was not possible. The procedure is illustrated in the flowchart presented (**Supplementary Figure S1**).

### Participants

Purposive sampling involving a direct approach was used to recruit participants to a panel of experts. Participants were invited via email through existing professional contacts known to the research team, as well as through relevant professional networks. The eligibility criteria for the current study were as follows: (a) the participants were members of MDTs supporting people with intellectual disabilities in the UK, and (b) involved in the process of optimising psychotropic medication in people with intellectual disabilities including psychotropic deprescribing. Care was taken to ensure the panel reflected the multi-disciplinary composition commonly observed in teams supporting individuals with intellectual disabilities and included registered learning disability nurses, psychiatrists (learning disability specialist), pharmacists, psychologists, speech and language therapists (SLTs), occupational therapists (OTs), physiotherapists, dieticians and arts therapists. The recruitment of professionals as panel members was informed by the Royal College of Psychiatrists Quality Network for Learning Disability (34).

Given the number of different health professionals with multiple areas of expertise, we planned to have a minimum sample size of 50 participants (45). Although the make-up of MDTs supporting people with intellectual disability can differ, core team members such as learning disability nurses and psychiatrists, were expected to compromise 60% and 80% of the panel, given their significant roles in medicines optimisation (46–48). Furthermore, we used “snowball sampling” to increase our sample by encouraging the pool of potential panel members to send invitations to other potential participants. In total 65 participants took part in round 1 and 62 participants in round 2. All participants in round 2 had previously participated in round 1. Whilst there is no consensus upon the number of participants required for a Delphi study, the panel size and composition were designed to ensure robust representation across professional groups within the MDT. The breakdown of professional representation on the panel in each round is presented in **Supplementary Table S2**.

### Round 1

In round 1, respondents were presented with a set of 34 statements (**Supplementary Table S3**) to be rated on a 5‐point Likert type scale from “unimportant” to “very important” with an additional “don’t know” option also provided. Each statement addressed a factor identified from previous research as contributing to optimal psychotropic deprescribing in people with intellectual disabilities. For clarity and ease of reference the statements were presented with overarching category labels, with typically two similar statements grouped together. At the end of the round 1 survey, respondents were invited to write a free text statement regarding any other factor involved in optimising the psychotropic deprescribing process that was not included in the survey. The Qualtrics^XM^ questionnaire remained open for 119 days and reminders to complete the survey sent to potential participants, who were yet to complete the questionnaire, at 2 weeks and at 4 weeks. Sixty-five responses were received in total.

### Round 2

The participants who had completed and provided consent in the first round were given access to the second round. Responses from the first round were collated and used to create the second-round questionnaire of 10 statements (**Supplementary Table S4**). Those statements that did not achieve a consensus level of ≥ 85% in round 1 were re-presented in round 2; participants were provided with feedback on their first-round responses to these statements by displaying their individual ratings relative to the overall ratings made by the whole panel for each statement. Four additional statements (**Supplementary Table S5**) developed from the analysis of the free text responses were also included in the second round, which remained open for 43 days. Participants were reminded by email when the questionnaire was approaching the date of closure at 2 and 4 weeks. Sixty-two responses were received with a second-round response rate of 95%.

### Data analysis

Qualtrics^XM^ was used for data collection and analysis. Consensus was measured by the percentage of respondents who rated a given item as “very important” or “extremely important”. There is no agreed standard for consensus levels within the Delphi literature (49). Those items that achieved a consensus level of ≥ 85% in rounds 1 and 2 were retained. Those items that did not receive the required consensus level in round 1 were re-presented in round 2. The free text data were exported to a word document and discussed amongst the research team to identify any missing factors optimising psychotropic deprescribing from our set of statements. At the completion of round 2, we divided the expert panel into three subgroups: psychiatrists (n= 19), nurses (n= 23 and others (n= 20) to explore any further consensus.

## Results

Following the round 1 questionnaire, twenty-eight statements reached the consensus criterion of ≥ 85% ratings being “very important” or “extremely important” (**Supplementary Table S6**).

Consensus was reached on all statements reflecting attitudes and confidence derived from our previous study that involved pharmacists (29). In addition, all statements reflecting conflict resolution, person-centred care, shared decision-making, adherence to guidelines and mutual learning and support, developed from the framework by Ghosh et al. (30), also achieved consensus.

Six statements did not achieve consensus of ≥ 85% in round 1 and were presented again to the panel in round 2 (**Supplementary Table S7**).

These statements lacking initial consensus focused upon collaborative, team-based, and structured approaches to psychotropic deprescribing for individuals with intellectual disabilities. However, the statements “Psychotropic deprescribing decisions discussed by a multi-disciplinary team rather than being taken solely by individual prescribers” and “The implementation of a specific appropriately resourced deprescribing programme” reached over 80% agreement. Furthermore, the statements “Active engagement by General Practitioners (GPs),” and “Providing continuity of care involving the same healthcare professional working with the individual with intellectual disabilities” achieved over 70% agreement. Two statements that were developed from the work completed by Ghosh et al. (30), “The implementation of a specific appropriately resourced deprescribing programme”, and “Providing continuity of care involving the same healthcare professional working with the individual with intellectual disabilities” did not achieve consensus.

Twenty-five participants provided free text data in round 1 which was exported from Qualtrics^XM^ to a word document available in Appendix 1. While the majority of the free text data were judged to duplicate existing statements, four additional statements, not previously identified within the literature, were developed and presented in round 2 (**Supplementary Table S5**).

Five statements reached consensus of ≥ 85% rating statements as “very” or “extremely important” following round 2 (**Supplementary Table S8**). Two of these statements, “High quality social care support in place for the person with intellectual disabilities,” and “Maintaining a positive culture towards psychotropic deprescribing across health and social care services” derived from the free text data in round 1, achieved over 90% consensus.

Five statements did not achieve consensus in the second round, three of which were previously presented in round 1. The statements “Active engagement by General Practitioners (GPs)” and “The clinical team includes non-medical prescribers with experience of intellectual disabilities” achieved over 70% agreement. Interestingly, consensus was achieved for “Active engagement by General Practitioners (GPs)” among the subgroup “others”. Consensus was also achieved for “The clinical team includes non-medical prescribers with experience of intellectual disabilities” among nurses. The statements developed from free text data, “A primary care deprescribing pathway in place” and “Training prescribers in other interventions such as art therapies, activity-based interventions or psychological interventions” did not reach 60% agreement.

The final list of 33 factors that were judged to support optimal psychotropic deprescribing are presented in **Table 1**. Twenty-one factors reached a consensus of 90% or higher and 8 factors reached a consensus of 100%. The factors are presented in descending order based on the percentage of participants rating them as important.

**Table 1:** Factors optimising psychotropic deprescribing.

	Factor optimising psychotropic deprescribing	Percentage agreement at the end of the study
1	Encouraging open communication between healthcare professionals and carers throughout the deprescribing process	100
2	Developing and maintaining strong partnerships between the multi-disciplinary team and individuals with intellectual disabilities	100
3	Developing and maintaining strong partnerships between the multi-disciplinary team and the carers of individuals with intellectual disabilities	100
4	Offering the person with intellectual disabilities a range of non-pharmacological interventions	100
5	Regular medication review involving physical health monitoring	100
6	Proactively addressing carers’ fears of potential negative consequences of deprescribing	100
7	Proactively addressing fears of people with intellectual disabilities of potential negative consequences of deprescribing	100
8	Healthcare professionals who are confident to initiate deprescribing	100
9	Encouraging open communication between healthcare professionals and the person with intellectual disabilities throughout the deprescribing process.	98
10	Providing the person with intellectual disabilities long term regular support throughout the deprescribing process	98
11	Prescribers have a specialist knowledge of prescribing psychotropic medicines in people with intellectual disabilities.	98
12	Reasonable and personalised adjustments to tailor the deprescribing process to the individual needs of the person with intellectual disabilities	98
13	Empowering people with intellectual disabilities to be fully included in decision-making.	98
14	Providing education about deprescribing to healthcare and social care professionals supporting people with intellectual disabilities	97
15	Having sufficient time to provide personalised reasonable adjustments for deprescribing	97
16	High quality social care support in place for the person with intellectual disabilities	97
17	People with intellectual disabilities attending an annual physical health check at their GP practice	95
18	Focussing on resolving disagreements between healthcare professionals and carers regarding the deprescribing process	95
19	Focussing on resolving disagreements between healthcare professionals and people with intellectual disabilities regarding the deprescribing process	95
20	Prescribers are experienced in providing healthcare to people with intellectual disabilities	95
21	Healthcare professionals having the confidence to motivate people with intellectual disabilities to engage in deprescribing psychotropic medicines.	92
22	Empowering carers to be fully included in decision-making	92
23	Maintaining a positive culture towards psychotropic deprescribing across health and social care services	92
24	Providing continuity of care involving the same healthcare professional working with the individual with intellectual disabilities	90
25	Healthcare professionals having the confidence to motivate carers to engage in deprescribing psychotropic medicines.	90
26	Effective implementation of Positive Behavioural Support	89
27	Healthcare professionals who are motivated to identify people with intellectual disabilities who may be suitable for deprescribing	89
28	The implementation of a specific appropriately resourced deprescribing programme	88
29	People with intellectual disabilities, their careers and healthcare professionals all learning from each other’s experiences	88
30	Healthcare professionals feeling valued by their clinical team in their efforts to deprescribe	87
31	A mix of individuals from different professional backgrounds involved with the psychotropic deprescribing process	87
32	Psychotropic deprescribing decisions discussed by a multi-disciplinary team rather than being taken solely by individual prescribers.	85
33	Adhering to evidence-based prescribing guidelines including those specifically focused on psychotropic deprescribing	85

## Discussion

The aim of this modified Delphi study was to achieve consensus among healthcare professionals working in MDTs supporting individuals with intellectual disabilities within secondary care services on factors that facilitate psychotropic deprescribing in people with intellectual disabilities.

The key finding was a set of 33 factors supporting optimal psychotropic deprescribing within the UK that achieved consensus. These factors related to multi-disciplinary team working, communication, person-centred care, shared decision-making, ongoing support, knowledge and experience, education and training needs, access to non-pharmacological interventions, and regular medication review. This represents a substantial proportion of the original set of facilitators, suggesting the modified Delphi questionnaire was robust and grounded in evidence (24, 29, 30).

While many factors were specific to deprescribing, others addressed broader aspects of medicines optimisation. Of the 33 factors that achieved consensus, 20 directly related to deprescribing, with “collaboration” identified as central to 12 of these. Among the 13 factors not specific to deprescribing, eight also related to collaboration which was an overarching theme in the conceptual framework published by Ghosh et al. (30).

Consensus was not reached on three factors from the original list including the importance of GP engagement and collaborative working with social workers which are both related to multi-agency working. Similarly, one factor identified in round 1, that did not achieve consensus, a primary care deprescribing pathway in place, is also related to multi-agency working. This lack of consensus may be due to the exclusion of GPs from our study and to the fact that we did not recruit social workers. However, Kelly et al. (50) found that GPs were less likely to engage in deprescribing due to fear of relapse with patients who had long-term and/or recurrent depression, older patients, and those with comorbidities. Considering those with intellectual disabilities, Shankar and Wilcock (51) identified that psychotropic medicines need to be managed by specialist care, as opposed to primary care. However, in routine NHS clinical practice psychotropic medicines may be initiated for individuals with intellectual disabilities by specialists in secondary care but typically it is GPs or non-medical prescribers within the GP surgery who continue to prescribe these medicines on a repeat basis and monitor their side effects. Furthermore there may be formal arrangements in place such as shared care agreements (52). Therefore, it is important that GPs actively engage with the psychotropic deprescribing process. Radcliffe et al. (53) found that collaboration between GPs and geriatricians can lead to more medicines being stopped and doses reduced suggesting that multi-agency working is helpful for deprescribing.

The third factor from the original list not to achieve consensus was the inclusion of non-medical prescribers with experience in intellectual disabilities as part of the clinical team. The second factor identified in round 1 that did not reach consensus was training prescribers in non-pharmacological interventions. Interestingly, both these factors reflect a move away from traditional prescribing roles. The lack of agreement on the latter may not necessarily reflect disagreement about the value of such approaches, but rather differing views on whether prescribers themselves need direct training, or whether they simply require greater awareness of existing supports and evidence-based interventions that can be accessed through other members of the multidisciplinary team.

The conceptual model developed by Ghosh et al. (30) did not include multi-agency working, non-medical prescribing, and availability and access to non-pharmacological interventions as factors for optimising medication in people with intellectual disabilities. Whilst the Ghosh model focused on medicines optimisation in general, our findings, which focus on deprescribing psychotropic medicines, aligned well within this framework.

### Implications for clinical practice

Our findings reinforce the importance of a team-based approach to deprescribing as opposed to decision-making by individual prescribers. A team-based approach is advantageous because it brings together diverse expertise, ensuring a holistic assessment of the individual’s medical, psychological, and social needs and the provision of robust non-pharmacological interventions. Such collaboration enhances the safety and effectiveness of deprescribing decisions with individuals with intellectual disabilities who often present with additional complexity.

Whilst consensus was reached about the importance of offering non-pharmacological interventions when attempting to optimise psychotropic deprescribing, there was lack of consensus about training prescribers in non-pharmacological interventions such as art therapies, activity-based interventions, or psychological interventions. This is unsurprising, considering that prescribers are unable to be specialists in all interventions, and further reinforces the importance of a team-based approach to deprescribing.

Consensus was reached about the importance of regular medication reviews and monitoring, including annual health checks at GP practices to help promote optimal psychotropic deprescribing. These checks are often conducted by a range of primary care professionals (54). However, whilst primary care is increasingly expected to play a greater role in deprescribing (55), our findings showed a lack of consensus regarding GP engagement and the potential value of a structured primary care deprescribing pathway.

### Strength and limitations

This research involved a range of panel experts with diversity of working within services for people with intellectual disabilities in the UK. We included the core professionals, namely nurses and psychiatrists, who have responsibility for prescribing and deprescribing. In addition, we included a range of other core healthcare professionals working within services for people with intellectual disabilities (i.e. registered psychologists, and registered allied health professions such as arts therapists, occupational therapists, speech and language therapists). We recruited professionals from across the UK to help maximise diversity of opinion and we exceeded our minimum sample size target.

However given the research design and despite achieving a high level of consensus, it is important to acknowledge limitations. First, the sample was limited to healthcare professionals within the UK, which may restrict the generalisability of the findings to other healthcare systems with different resources, practices and professionals. It is important to note that nurses and psychiatrists with specialist training and registration that enables them to work with people with intellectual disabilities are unique to the UK and Ireland. These roles do not exist in the same way in other countries. Second, whilst the modified Delphi method allowed for the inclusion of diverse perspectives, these perspectives are nevertheless subjective opinion and biased. While every effort was made to phrase statements clearly and concisely, free-text responses indicated that some participants may have interpreted certain terms differently. Furthermore, we did not recruit any social care professionals. The views of GPs, and the views of carers and individuals with intellectual disabilities themselves were not captured in this study representing a significant limitation given the potential importance of primary care and the importance of person-centred care and lived experiences in informing deprescribing practices. Finally, whilst the Delphi method is useful for building consensus, there was a relatively low level of discrimination across the statements, possibly limiting the ability to clearly prioritise the most important enablers of effective psychotropic deprescribing practice in people with intellectual disabilities. Additionally, variation in consensus, particularly for factors that did not reach the 85% agreement threshold, may reflect differences in professional role, training, or perspective. This highlights the potential influence of disciplinary background on the perceived importance of specific deprescribing factors.

### Future research

Future research should include the voice of people with intellectual disabilities and their carers to build on our findings and ensure a comprehensive, person-centred and inclusive understanding of factors influencing psychotropic deprescribing. There should be a focus to ensure that deprescribing practices are equitable, sustainable and grounded in lived experiences. Furthermore, future studies could explore the impact of factors such as ethnicity, heritage, and socioeconomic status on psychotropic deprescribing. These social determinants of health may interact with the identified factors in complex ways influencing access to care and the successful implementation of deprescribing interventions (56). As the emphasis of medication reviews looks to shift from secondary care to primary care, research studies involving GPs and other healthcare professionals working in primary care settings would be very valuable to develop safe and effective psychotropic deprescribing practices. To fully support psychotropic deprescribing in primary care further work is needed to clarify the interface between primary care and specialist intellectual disability services and explore how they can collaborate more effectively to integrate structured deprescribing pathways into routine practice, including within annual health checks. Key priorities should include developing clear shared care protocols, improving communication channels between services, and ensuring role clarity around responsibilities for initiating, reviewing, and monitoring psychotropic medication changes. In addition, future research should explore prescribers’ awareness of and access to evidence-based non-pharmacological supports when considering deprescribing. Future research could also build upon our study by testing the implementation of the agreed set of facilitators in real-world settings using feasibility and implementation studies, evaluating their impact on patient outcomes, and exploring how they can be adapted to different healthcare settings and systems.

## Data Availability

The original contributions presented in the study are included in the article/**Supplementary Material**. Further inquiries can be directed to the corresponding author.
