# Supplementary material for: Factors supporting optimisation of psychotropic deprescribing in people with intellectual disabilities within the UK: a modified Delphi study
Source: Front Psychiatry. 2025 Aug 19;16:1652988. doi: 10.3389/fpsyt.2025.1652988 (PMC12402727; doi:10.3389/fpsyt.2025.1652988)
Supplement: Supplementary file 1 [file DataSheet1.pdf]

Appendix 1 Delphi study Psychotropic Deprescribing

Free text responses

1. Factoring in the attitudes, beliefs and feelings of staff and carers towards medication as a predictor safe and effective deprescribing outcomes (applied behaviourists might call this the 'contextual fit' for a deprescribing intervention).
2. Preparing service-users, staff and carers for negative withdrawal/rebound effects of deprescribing, as something to be worked through and managed, rather than automatically signal a failed deprescribing attempt.
3. That weight and physical health biomarkers are monitored
4. The human rights aspect does not come across strongly in this study. The most powerful statement i had was "would you take it? No, so why are you giving it to someone who has difficulties in communicating with you". Also the additional cognitive effect. My most successful deprescribing was seizure prevention drugs. Risk assessment and management tools were very important. Education is also essential. Long term use of psychotropic medication results in premature death. End of.
5. Role of Education/Short breaks/Respite/Social opportunities for children and young people and their involvement in the process
6. Ensuring a follow up date is in place at the end of the consultation to provide reassurance of ongoing review
7. Education to current prescribers such as consultant psychiatrists and GPs. Sharing success stories widely to encourage others and reduce fears.
8. Training for prescribers around learning disabilities, alternative therapeutic therapies. A clear Primary care Deprescribing pathway, the importance to look at other factors that may in pack on the success of the medication reduction.
9. All of the questions raised in this survey are important. We however must remember that not everyone with a LD can engage in their care in the way described and so this is where the relationship with carers is paramount (which possibly could have been made clearer in some of your questions). It is also important that people with a LD are empowered in all aspects of their care where ever possible to make their own decisions.
10. Adequate social support including care provision and activities.
11. Adequate resourcing of non-pharmacological alternatives, primarily for family/social care providers to upskilled to be able to effectively implement positive behaviour management strategies. Correct implementation of the Mental Capacity Act in relation to assessment of the patient's capacity to consent to treatment/deprescribing protocol.
12. Deprescribing is very individual, the preferred approach may not be applicable to all.
13. It is important to be aware that prescribing and de-prescribing is a dynamic issue and a "culture". This cultural change needs to be systematic with outcome measures built to measure change over time.
14. MDTs including and promoting non-pharmacological disciplines such as psychology, arts therapies and PBS as the first steps and these teams and their work being understood by prescribers.
15. I think the patients wishes are central to all of us wherever the patient has capacity. Some patients are motivated towards a deprescribing approach, whereas others feel quite strongly that they do not wish to have their medications reduced. We should always listen and take their views on board too, rather than unilaterally imposing a plan.
16. Provision of psychoeducation on restrictive practice as part of the process
17. Resources - need more health care practitioners to support deprescribing

18. Support team and/or family dynamics where people are supported round the clock by more than one person; its importnant all understand what and why to ensure no one sabotages the plan
19. you may have included it in the reasonable adjustment but I would say providing information in easy read format is extremely important
20. Carers attending appointments with required information (including concrete info about concerns on what might happen if deprescribing)
21. Some deprescribing is historical. Whilst knowledge of psychotropics is important, such deprescrbing can be delivered by prescribers who perhaps are not experts in LD and the deprescribing, but have support from someone who is. We must not forget this, in an environment where expertise may be thin on the ground. Often in historical cases, PBS or other alternative interventions are not required when deprescribing is initiated, as the medication as been taken for years and patients/carers/HCPs aren't sure why the medicine was started in the first place!
22. The focus should be on 'evidence based prescribing'. That will naturally involve de-prescribing when appropriate. The repeated emphasis on 'de-prescribing' as an end by itself risks drifting into an ideological, rather than evidence-based position.
23. Availability of medications in suitable forms to allow for safe deprescribing (e.g. liquid forms so a slower reduction can take place if needed).
24. Make sure have a good history if possible on why medication started and if there is a history of mental illness
25. Individuals being in the right environment with appropriately trained carers attuned to their needs, is hugely significant in the success of the de-prescribing process
