# Supplementary material for: Factors supporting optimisation of psychotropic deprescribing in people with intellectual disabilities within the UK: a modified Delphi study
Source: Front Psychiatry. 2025 Aug 19;16:1652988. doi: 10.3389/fpsyt.2025.1652988 (PMC12402727; doi:10.3389/fpsyt.2025.1652988)
Supplement: Supplementary file 2 [file DataSheet2.pdf]

Supplementary Information

Table S1

Delphi studies in social and health sciences – recommendations for an interdisciplinary standardized reporting (DELPHISTAR).

This reporting guideline is meant for studies using Delphi techniques in the health and social sciences. These also include all Delphi variants and modifications that meet the following criteria:

1. Survey of several people with specialized knowledge (e.g., operational knowledge, experiential knowledge, functional knowledge, contextual knowledge);
2. Structured communication process that involves a group of people with relevant expertise;
3. Carrying out at least two survey rounds or the option to respond at least two times;
4. Feedback: the (interim) results are presented to the respondents starting in the second round;
5. Basis is a quantitative questionnaire with the possibility to contribute or supplement arguments for the respective position;
6. All answers, quantitative and qualitative, are systematically analyzed (quantitative: e.g., descriptive statistics, qualitative: e.g., thematic analysis).

Topic	Section	Item	Checklist Item	Location where item is reported	Exemplary answer
I Title and Abstract		1	Identification as a Delphi procedure in the title		Factors associated with optimising psychotropic deprescribing in people with intellectual disabilities: a Delphi Exercise
		2	Identification as a Delphi procedure in the abstract		A Delphi procedure was selected to answer the research question.
		3	Structured abstract		background, method, results and discussion
II Context	Formal	4	Information about the sources of funding		This Delphi received no funding
		5	Information about the team of authors and/or researchers (e.g., discipline, institution)		The Delphi study was conducted by a doctoral research team including a PhD student and three academic professors
		6	Information about method consulting		No outside consulting in regard to method took place.
		7	Information about the project background		The Delphi survey was part of a PhD research project on deprescribing psychotropic medicines in people with intellectual disabilities
		8	Information about the study protocol		The study protocol is available as supplementary data on request from author
	Content	9	Justification of the chosen method (Delphi procedure) to answer the research question		The Delphi method is suitable for answering the research question because it systematically gathers the judgments of a range of healthcare professionals and can identify agreement and disagreement.
		10	Aim of the Delphi procedure (e.g., consensus, forecasting)		The aim of the Delphi study is to find consensus on a range of enablers of psychotropic deprescribing
III Method	Body & Integration of knowledge	11	Identification and elucidation of relevant expertise, spheres of experience, and		The experts represent the composition of MDT healthcare teams supporting people with intellectual disabilities throughout the UK.

Topic	Section	Item	Checklist Item	Location where item is reported	Exemplary answer
			perspectives (e.g., theory, practice, affected groups, disciplines)		
		12	Handling of knowledge, expertise and perspectives which are missing or have been deliberately not integrated		N/A
		13	Basic definition of expert[1]		A person who is working in MDT healthcare teams supporting people with intellectual disabilities throughout the UK and contributes towards the psychotropic deprescribing process in people with intellectual disabilities is considered to be an expert.<br><br>This contribution includes not only prescribing roles, but may also include activities such as advising people with intellectual disabilities and carers about medication effects, reviewing and supporting with any psychological or behavioural concerns during medication changes, and advocating for medication reviews when liaising with other healthcare professionals.
	**Delphi variations**	14	Identification of the type of Delphi procedure and potential modifications (e.g., classic Delphi, real-time Delphi, group Delphi)		Modified Delphi – refer to the paper for details
		15	Justification of the Delphi variation and modifications, including during the Delphi process, if applicable		Previous studies informed the development of the questionnaire and therefore a first qualitative round was not necessary
	**Sample of experts**	16	Selection criteria for the experts (per round if there are different expert groups)		All of the experts who met the definition were invited to the first round.<br>All of the experts who completed the previous round were invited to participate in the subsequent round.
		17	Identification of the experts		The experts were identified based on personal contacts of the research team plus professional organisations such as Radiant and learning disability practitioner group.
		18	Information about recruiting and any subsequent recruiting of experts		The experts were informed about the Delphi study and invited to participate.
	**Survey**	19	Elucidation of the content development for the questionnaire[2]		The questionnaire was developed based on (1) the framework describing factors supporting medicines optimisation in people with intellectual disabilities developed as part of a NIHR funded systematic review ref , (2) our findings from our previously completed systematic review about psychotropic deprescribing in this area13, 14 and (3) our previously completed survey of pharmacists'

Topic	Section	Item	Checklist Item	Location where item is reported	Exemplary answer
					experiences of deprescribing psychotropic medicines for this patient population ref.
		20	Description of the questionnaire (content and structure)		The statements made in the questionnaire were rated using standardized items, with the option for comment in one final free-text box
	**Delphi rounds**	21	Number of Delphi rounds		Two Delphi rounds were held.
		22	Information about the aims of the individual Delphi rounds		Standard evaluation
		23	Disclosure and justification of the criterion for discontinuation		The number of rounds was defined in advance to be a maximum of three rounds.
	**Feedback**	24	Information about what data was reported back per round		In terms of feedback, we shared the personal results with each individual expert plus the overall results for the panel. We only shared the results of the statements not achieving consensus after round 1.
		25	Information on how the results of the previous Delphi round were fed back to the experts surveyed (e.g., via frequencies, mean values, measures of dispersion, listing of comments)		The results were fed back as percentages scores
		26	Information on whether feedback was differentiated by specific groups (e.g., by field of expertise, institutional affiliation)		The feedback was aggregated across all expert groups.
		27	Information about how dissent and unclear results were handled		The results showing dissent were presented again for evaluation in the next Delphi round.
	**Data analysis**	28	Disclosure of the quantitative and qualitative analytical strategy		The quantitative items were descriptively analyzed. The open-ended items were analyzed through research group discussion and content analysis.
		29	Definition and measurement of consensus		Consensus was defined as percentage agreement, meaning that agreement was assumed if at least 85% of the respondents rated an item either "very important" or "extremely important"
		30	Information on group-specific analysis or weighting of experts (e.g., theory vs. practice, discipline-specific analysis)		Specific analysis for individual healthcare professionals was carried out for statements not achieving consensus
**IV Results**	**Delphi process**	31	Illustration of the Delphi process (e.g., in a flow chart)		A summary of the process is illustrated in a flow chart (Figure 1).
		32	Information about special aspects during the Delphi process (e.g., deviations from the intended approach with justification)		N/A

Topic	Section	Item	Checklist Item	Location where item is reported	Exemplary answer
		33	Number of experts per round (both invited and participating)		The number of experts participating in the first Delphi round was 65 and the number of experts in the second round was 59.
	**Results**	34	Presentation of the results for each Delphi round and the final results		In both Delphi rounds 85% or more of the respondents agreed
**V Discussion**	**Quality of findings**	35	Highlighting the findings from the Delphi study		The central findings can be summarized as follows: This Delphi exercise has provided a list of factors optimising psychotropic deprescribing in people with intellectual disabilities as endorsed by healthcare teams.
		36	Validity of the results (e.g., transferability of the findings)		Findings are transferable to other countries although some findings will not be relevant to healthcare systems in other countries
		37	Reliability of the results (e.g., split half, inter-rater reliability)		The responses in the free-text comments box were analyzed by the research team
		38	Reflection on potential limitations (e.g., distortion, skewing, bias)		The results are to be viewed critically with regard to sample limited to UK, reliance on self reported ratings and inability to recruit social workers. GPs, people with intellectual disabilities and carers were not eligible to participate in this study

[1] "Experts" are the participants; this can be people from academia, practice, or representatives of lived experience (e.g., patients, family members).
[2] The term "questionnaire" stands for the survey instrument regardless of whether quantitative or qualitative items are integrated or weighted.

Contact

Prof. Dr. Marlen Niederberger
E-mail: marlen.niederberger(at)ph-gmuend.de
Department of Research Methods in Health Promotion and Prevention, Institute for Health Sciences
University of Education Schwäbisch Gmünd, Oberbettringer Straße 200, 73525 Schwäbisch Gmünd, Germany

Figure S1 Delphi Flowchart

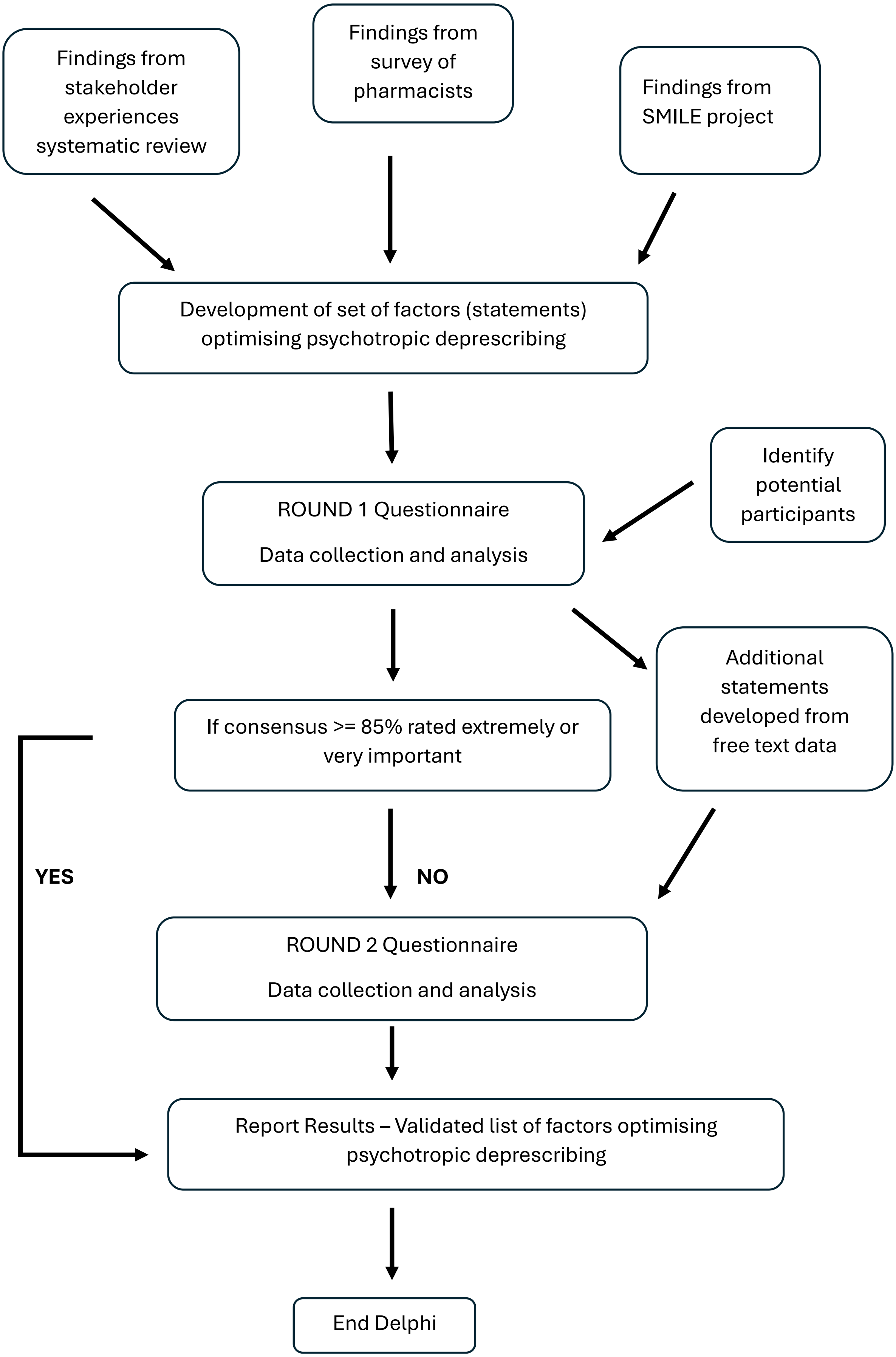

Table S2

The professional representation of the expert panel

Professional Group	Number in Round 1	Number in Round 2
Psychiatrist	19	18
Learning Disability Nurse	23	23
Pharmacist	6	5
Psychologist	4	4
Occupational Therapist	3	3
Speech and Language Therapist	5	4
Arts Therapist	3	3
Physiotherapist	1	1
Dietician	1	1

Table S3

Set of statements presented to the panel in round 1

	**Multi-disciplinary Team Working**
1	A mix of individuals from different professional backgrounds involved with the psychotropic deprescribing process
2	Psychotropic deprescribing decisions discussed by a Multi-Disciplinary Team rather than being taken solely by individual prescribers.
	**Multi-Agency Working**
3	Active engagement by General Practitioners (GPs)
4	Collaborative working with social workers
	**Communication**
5	Encouraging open communication between healthcare professionals and the person with intellectual disabilities throughout the deprescribing process.

6	Encouraging open communication between healthcare professionals and carers throughout the deprescribing process
	**Building Relationships with carers and people with intellectual disabilities**
7	Developing and maintaining strong partnerships between the Multi-Disciplinary Team and individuals with intellectual disabilities
8	Developing and maintaining strong partnerships between the Multi-Disciplinary Team and the carers of individuals with intellectual disabilities
	**Person Centred Care**
9	Reasonable and personalised adjustments to tailor the deprescribing process to the individual needs of the person with intellectual disabilities
	**Shared Decision Making**
10	Empowering people with intellectual disabilities to be fully included in decision making.
11	Empowering carers to be fully included in decision making
	**Providing Ongoing Support**
12	Providing the person with intellectual disabilities long term regular support throughout the deprescribing process
13	Providing continuity of care involving the same healthcare professional working with the individual with intellectual disabilities.
	**Mutual Learning and Support**
14	People with intellectual disabilities, their carers and healthcare professionals all learning from each other’s experiences
	**Adherence to Treatment Guidelines and Protocols**
15	Adhering to evidence-based prescribing guidelines including those specifically focused on psychotropic deprescribing
	**Non Medical Prescribers**
16	The clinical team includes non-medical prescribers with experience of intellectual disabilities
	**Availability and access to non-pharmacological interventions in addition to medication**
17	Offering the person with intellectual disabilities a range of non-pharmacological interventions

18	Effective implementation of Positive Behavioural Support
	**Regular Medication Review and Monitoring**
19	Regular medication review involving physical health monitoring
20	People with intellectual disabilities attending an annual physical health check at their GP practice
	**Addressing fear of negative consequences following psychotropic deprescribing**
21	Proactively addressing carers' fears of potential negative consequences of deprescribing
22	Proactively addressing fears of people with intellectual disabilities of potential negative consequences of deprescribing
	**Resolving Conflict**
23	Focussing on resolving disagreements between healthcare professionals and carers regarding the deprescribing process
24	Focussing on resolving disagreements between healthcare professionals and people with intellectual disabilities regarding the deprescribing process
	**Knowledge and Experience**
25	Prescribers have a specialist knowledge of prescribing psychotropic medicines in people with intellectual disabilities.
26	Prescribers are experienced in providing healthcare to people with intellectual disabilities
	**Education and Training Needs**
27	Providing education about deprescribing to healthcare and social care professionals supporting people with intellectual disabilities
	**Workload and Resources**
28	Having sufficient time to provide personalised reasonable adjustments for deprescribing
29	The implementation of a specific appropriately resourced deprescribing programme
	**Attitudes**
30	Healthcare professionals who are motivated to identify people with intellectual disabilities who may be suitable for deprescribing

31	Healthcare professionals feeling valued by their clinical team in their efforts to deprescribe
	**Confidence**
32	Healthcare professionals having the confidence to motivate people with intellectual disabilities to engage in deprescribing psychotropic medicines.
33	Healthcare professionals having the confidence to motivate carers to engage in deprescribing psychotropic medicines.
34	Healthcare professionals who are confident to initiate deprescribing

Table S4

Set of statements presented to the panel in round 2

	**Statement**
1	Psychotropic deprescribing decisions discussed by a Multi-Disciplinary Team rather than being taken solely by individual prescribers.
2	Active engagement by General Practitioners (GPs)
3	Collaborative working with social workers
4	Providing continuity of care involving the same healthcare professional working with the individual with intellectual disabilities.
5	The clinical team includes non-medical prescribers with experience of intellectual disabilities
6	The implementation of a specific appropriately resourced deprescribing programme
7	High quality social care support in place for the person with intellectual disabilities
8	Maintaining a positive culture towards psychotropic deprescribing across health and social care services

9	A primary care deprescribing pathway in place
10	Training prescribers in other interventions such as art therapies, activity-based interventions or psychological interventions

Table S5

Statements developed from free text data

1	High quality social care support in place for the person with intellectual disabilities
2	Maintaining a positive culture towards psychotropic deprescribing across health and social care services
3	A primary care deprescribing pathway in place
4	Training prescribers in other interventions such as art therapies, activity-based interventions or psychological interventions

Table S6

Statements achieving consensus in round 1

	**Statement**	**Percentage agreement**
1	A mix of individuals from different professional backgrounds involved with the psychotropic deprescribing process	87
2	Encouraging open communication between healthcare professionals and the person with intellectual disabilities throughout the deprescribing process.	98
3	Encouraging open communication between healthcare professionals and carers throughout the deprescribing process	100

4	Developing and maintaining strong partnerships between the Multi-Disciplinary Team and individuals with intellectual disabilities	100
5	Developing and maintaining strong partnerships between the Multi-Disciplinary Team and the carers of individuals with intellectual disabilities	100
6	Reasonable and personalised adjustments to tailor the deprescribing process to the individual needs of the person with intellectual disabilities	98
7	Empowering people with intellectual disabilities to be fully included in decision making.	98
8	Empowering carers to be fully included in decision making	92
9	Providing the person with intellectual disabilities long term regular support throughout the deprescribing process	98
10	People with intellectual disabilities, their carers and healthcare professionals all learning from each other's experiences	88
11	Adhering to evidence-based prescribing guidelines including those specifically focused on psychotropic deprescribing	85
12	Offering the person with intellectual disabilities a range of non-pharmacological interventions	100
13	Effective implementation of Positive Behavioural Support	89
14	Regular medication review involving physical health monitoring	100
15	People with intellectual disabilities attending an annual physical health check at their GP practice	95
16	Proactively addressing carers' fears of potential negative consequences of deprescribing	100
17	Proactively addressing fears of people with intellectual disabilities of potential negative consequences of deprescribing	100
18	Focussing on resolving disagreements between healthcare professionals and carers regarding the deprescribing process	95
19	Focussing on resolving disagreements between healthcare professionals and people with intellectual disabilities regarding the deprescribing process	95
20	Prescribers have a specialist knowledge of prescribing psychotropic medicines in people with intellectual disabilities.	98

21	Prescribers are experienced in providing healthcare to people with intellectual disabilities	95
22	Providing education about deprescribing to healthcare and social care professionals supporting people with intellectual disabilities	97
23	Having sufficient time to provide personalised reasonable adjustments for deprescribing	97
24	Healthcare professionals who are motivated to identify people with intellectual disabilities who may be suitable for deprescribing	89
25	Healthcare professionals feeling valued by their clinical team in their efforts to deprescribe	87
26	Healthcare professionals having the confidence to motivate people with intellectual disabilities to engage in deprescribing psychotropic medicines.	92
27	Healthcare professionals having the confidence to motivate carers to engage in deprescribing psychotropic medicines.	90
28	Healthcare professionals who are confident to initiate deprescribing	100

Table S7

Statements not achieving consensus in round 1

	**Statement**	**Percentage agreement**
1	Psychotropic deprescribing decisions discussed by a Multi-Disciplinary Team rather than being taken solely by individual prescribers.	84
2	Active engagement by General Practitioners (GPs)	71
3	Collaborative working with social workers	56

4	Providing continuity of care involving the same healthcare professional working with the individual with intellectual disabilities.	79
5	The clinical team includes non-medical prescribers with experience of intellectual disabilities	61
6	The implementation of a specific appropriately resourced deprescribing programme	81

Table S8

Statements achieving consensus in round 2

	**Statement**	**Percentage agreement in round 2**
1	Psychotropic deprescribing decisions discussed by a Multi-Disciplinary Team rather than being taken solely by individual prescribers.	85
2	Providing continuity of care involving the same healthcare professional working with the individual with intellectual disabilities	90
3	The implementation of a specific appropriately resourced deprescribing programme	88
4	High quality social care support in place for the person with intellectual disabilities	97
5	Maintaining a positive culture towards psychotropic deprescribing across health and social care services	92
